# Running wheel exercise reduces α-synuclein aggregation and improves motor and cognitive function in a transgenic mouse model of Parkinson's disease

**DOI:** 10.1371/journal.pone.0190160

**Published:** 2017-12-22

**Authors:** Wenbo Zhou, Jessica Cummiskey Barkow, Curt R. Freed

**Affiliations:** Division of Clinical Pharmacology and Toxicology, Departments of Medicine, Pharmacology, Neurology, and Neurosurgery; University of Colorado Denver, School of Medicine, Aurora, CO, United States of America; Thomas Jefferson University, UNITED STATES

## Abstract

Exercise has been recommended to improve motor function in Parkinson patients, but its value in altering progression of disease is unknown. In this study, we examined the neuroprotective effects of running wheel exercise in mice. In adult wild-type mice, one week of running wheel activity led to significantly increased DJ-1 protein concentrations in muscle and plasma. In DJ-1 knockout mice, running wheel performance was much slower and Rotarod performance was reduced, suggesting that DJ-1 protein is required for normal motor activity. To see if exercise can prevent abnormal protein deposition and behavioral decline in transgenic animals expressing a mutant human form of α-synuclein in all neurons, we set up running wheels in the cages of pre-symptomatic animals at 12 months old. Activity was monitored for a 3-month period. After 3 months, motor and cognitive performance on the Rotarod and Morris Water Maze were significantly better in running animals compared to control transgenic animals with locked running wheels. Biochemical analysis revealed that running mice had significantly higher DJ-1, Hsp70 and BDNF concentrations and had significantly less α-synuclein aggregation in brain compared to control mice. By contrast, plasma concentrations of α-synuclein were significantly higher in exercising mice compared to control mice. Our results suggest that exercise may slow the progression of Parkinson’s disease by preventing abnormal protein aggregation in brain.

## Introduction

Parkinson’s disease is characterized by the loss of midbrain dopamine neurons in the substantia nigra pars compacta [1, 2]. Death of dopamine neurons has been attributed to oxidative stress, abnormal protein aggregation, and genetic factors [3–5]. Mutations in many genes have been linked to Parkinson’s including α-synuclein, Parkin, UCHL1, DJ-1, PINK1, LRRK2, and VSP35 [6–8]. The gene mutations could lead to either loss of neuroprotective functions such as DJ-1 and PINK1, or gain of toxic functions such as α-synuclein and LRRK2 [9].

Single amino acid mutations in the α-synuclein gene as well as simple triplication of the gene have been shown to cause autosomal-dominant forms of Parkinson’s disease [10, 11]. Interestingly, α-synuclein has been found to be a major component of Lewy bodies which are pathological hallmarks for idiopathic Parkinson’s disease [12, 13]. Transgenic mice overexpressing human wild-type or A53T mutant α-synuclein have recapitulated many features of Parkinson’s disease, such as impaired motor function, abnormal protein aggregation, and neuronal degeneration [14–17]. Monomeric α-synuclein protein is highly soluble; however, it can easily aggregate under various conditions such as low pH, high concentration, presence of metal ions, and oxidative stress. The formation of insoluble α-synuclein fibrils involves several intermediate species, such as dimers, oligomers, and protofibrils. Recent evidence suggests that α-synuclein oligomers are the most neurotoxic form of α-synuclein protein, and oligomer secretion is critical for the spreading and progression of Parkinson’s neuropathology [18–20]. Therefore, preventing α-synuclein aggregation could provide a major therapeutic advance [21, 22].

DJ-1 is one of the Parkinson-associated genes in which mutations lead to early-onset, autosomal recessive disease. Because the loss of gene expression causes disease, the DJ-1 gene can be seen as protecting nearly everyone from developing Parkinson’s disease [23–25]. DJ-1 or its homologs are present in all life forms that use oxygen including all animals, all plants that perform photosynthesis, and all aerobic bacteria [26–29]. This critical gene protects cells by antioxidant mechanisms such as stabilizing Nrf2 (nuclear factor erythroid 2-related factor) and thereby upregulating a family of antioxidant response element (ARE) genes [30–32]. DJ-1 is also involved in regulating HIF1 transcriptional activity under hypoxic conditions [33]. We have shown that DJ-1 also protects cells from abnormal protein aggregation by upregulating Hsp70 [34, 35].

Because Parkinson’s disease leads to disabling bradykinesia and rigidity, exercise and physical therapy are often prescribed by physicians. The hope has been that exercise will enhance mobility, preserve muscle tone, and prevent medical complications such as pneumonia that are associated with immobility. Several clinical trials have found that regular exercise or physical therapy may improve motor function in Parkinson patients [36–39]. In acute, drug-induced animal models of Parkinson’s disease, exercise can partially protect dopamine neurons from neurotoxicity [40–45]. For Alzheimer’s disease, exercise in transgenic mouse models have shown improvement in cognitive function and reduction in β-amyloid deposition as well as other biochemical markers [46–50]. However, in transgenic mouse models of Huntington’s disease, results of exercise testing are conflicting; either accelerating disease progression [51] or partially improving motor and cognitive function [52–54]. In this report, we have found that running wheel exercise can be neuroprotective in transgenic mice which have a progressive, age-related form of a Parkinson’s-Plus, diffuse Lewy body disease.

## Materials & methods

### Animals

All animal procedures were approved by the Institutional Animal Care and Use Committee (IACUC) at the University of Colorado Denver. Mice with DJ-1 gene deletion (B6.129-*Park7*^tm1Mak^, abbreviated as DJ-1 KO mice) were kindly provided by Dr. Tak Mak (University of Toronto) [55]. The homozygous DJ-1 KO mice and wild-type C57BL/6J littermates were produced by breeding male and female heterozygous DJ-1 KO mice. The Y39C human α-synuclein transgenic mice (FVB-Tg(Thy1-SNCA*Y39C)5Crf, abbreviated as Y39C a-Syn Tg mice) have been described previously [56]. The animals express a human mutant form of α-synuclein (Y39C) in all neurons under control of the Thy-1 promoter and so are a model of diffuse Lewy body disease, a Parkinson-Plus disorder. The Y39C a-Syn Tg mice were bred through male heterozygous Y39C Tg mice with wild-type FVB/N female mice. Heterozygous Y39C Tg mice and wild-type FVB/N littermates were used for this study. For all running wheel studies, mice were individually housed in each cage with free access to food and water. For all experiments, 5 to 7 animals were used for each group. We tried to use both males and females equally if possible for each group.

### Running wheel exercise in mice

Animals were singly housed in cages with free access to a running wheel (12 cm in diameter) mounted on the food bin. Daily running distances were recorded with a bicycle odometer attached to the running wheel. Before testing the Y39C-human mutant α-synuclein transgenic strain, we first tested 6-month old FVB/N wild-type mice for one week to evaluate the distance traveled by normal mice having access to a running wheel. There were 5 wild-type mice each for the running group and for the control group, with 3 males and 2 females in each group. To see the effect of the DJ-1 gene on locomotor activity, we then tested mice with the DJ-1 gene knocked out (6 DJ-1 KO mice, 3 males and 3 females) and compared the distances they ran with wild-type C57BL/6 littermates (6 wild-type controls, 3 males and 3 females). Both groups were 10-months of age. These animals had access to running wheels for two weeks. For our Y39C human mutant α-synuclein transgenic mice, we tested 12-month old mice for their ability to exercise on running wheels for one week. A total of fourteen Y39C Tg mice were rank-ordered by their running distance during that one week and were assigned to long term exercise group (seven Y39C Tg mice, 4 males and 3 females) or to non-exercise group (seven Y39C Tg mice, 4 males and 3 females) in alternating rank order. The exercise group had free access to running wheels in each of their cages for three months, while the non-exercise group had locked, non-functioning running wheels in their cages. Weekly running wheel distances were recorded using a bicycle odometer for each exercising animal.

### Rotarod test

Mice were tested for their ability to run on a 3 cm diameter rotating rod (Rotarod) at speeds ranging from 3 to 33 rpm [35, 56]. Before the test, mice were trained to stay on the Rotarod at 3 rpm. During 5 testing days, mice were placed on the rotating rod at one of the pre-set speeds of 7, 14, 21, 28, or 33 rpm for a 5-minute trial. Each animal received 3 trials with 5-minute rest intervals between trials. The time the mice spent on the Rotarod without falling was recorded for each trial.

### Morris water maze testing

Spatial learning was assessed using the Morris Water Maze in our campus animal behavioral core [35, 56]. The maze included a circular tank (120 cm in diameter) filled to 10 cm below the edge of the tank with 27°C water that was made opaque by the addition of non-toxic black ink. A circular escape platform (10 cm in diameter) was located 1 cm below the surface of the water in a constant location in the northwest quadrant of the tank. Mice were first acclimated to the maze during three trial habituation sessions. Each testing session consisted of 4 consecutive days with four trials per day. The platform was invisible in the pool, and mice were allowed to swim for 60 seconds before being returned to the home cage. The time to find the platform from all training and testing sessions was collected.

### Open field testing

Mice were placed in a 1-meter square plastic box for 30 minutes of open field exploratory testing. The field was divided into central and peripheral areas. The exploratory paths were recorded for each animal via video camera and computer. The time that mice spent in central and peripheral areas was analyzed. The rearing events of animals were also recorded.

### Western blotting

The mouse brain and muscle tissues were dissected and homogenized in dissociation buffer with protease inhibitors [35, 56]. Blood plasma was prepared as described below. Protein concentrations were determined by the BCA method. Fifty μg of protein was separated on 10% SDS-PAGE gel and transferred to a nitrocellulose membrane. The blots were probed with antibodies to DJ-1 (1:5000, #AB9718, Millipore), α-synuclein (Syn-1, 1:2000, #610787; BDBioscience) and α-synuclein (LB509, 1:2000, #MABN824, Millipore), Hsp70 (1:2000, #AB9920, Millipore), BDNF (1:2000, #SAB2108004, Sigma), β-actin (1:4000, #A2228, Sigma), and mouse serum albumin (1:2000, #PA1-30899, Thermo Fisher Scientific). Blots were incubated with HRP-conjugated secondary antibodies (1:10,000; #115-035-003, #111-035-003, Jackson Immuno Research), followed by chemiluminescent detection. Protein densities were quantified by ImageJ software after scanning into image files and normalized to β-actin or mouse albumin.

### ELISA

Mouse blood was collected immediately after sacrifice through transcardiac needles and syringes containing EDTA as an anti-coagulant. The blood was centrifuged at 2000 rpm for 5 min. After centrifugation, the plasma fraction was collected and stored at -80°C. Plasma was used to determine DJ-1 concentrations using an ELISA kit (CircuLex), according to manufacturer’s instructions.

### Statistics

For behavioral tests and biochemical analyses, there were 5–7 animals per group. The number of animals for each group was determined by power analysis using our previous behavioral and biochemical data. Data were expressed as mean ± SEM. Data were analyzed using t-test or ANOVA test followed by the Fisher LSD post hoc test.

## Results

### Exercise effects on DJ-1 levels in muscle and plasma of wild-type mice

**Fig 1:**
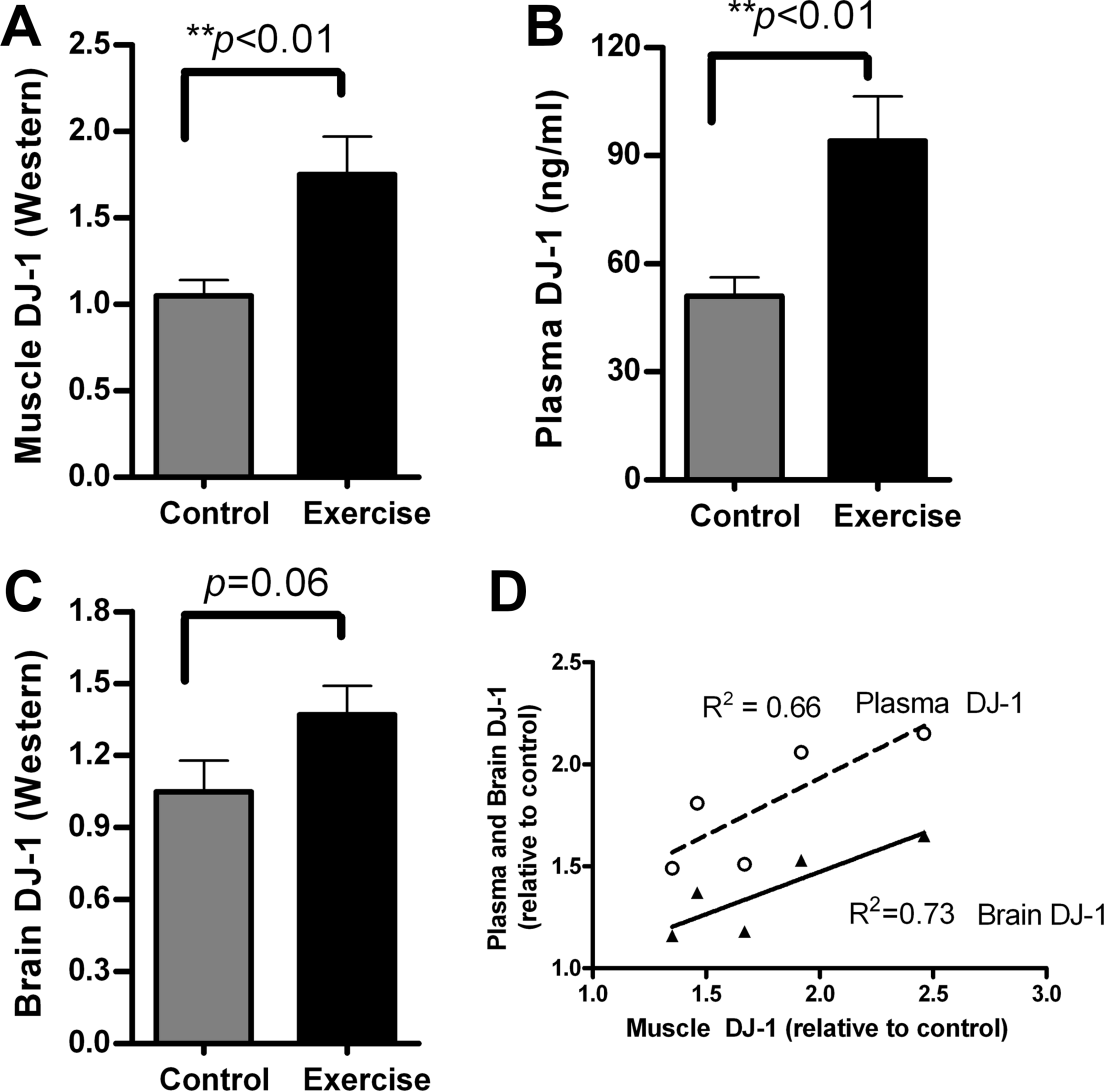
Running wheel exercise in wild-type mice increased DJ-1 expression in muscle, plasma, and brain. Adult FVB/N wild-type mice (4–6 months old) were assigned to either an exercise group or a control group (n = 5 each). After one week of running wheel exercise, animals were sacrificed and tissues collected for DJ-1 protein analysis by Western blot (muscle and brain) and ELISA (plasma). (**A**) Quantitative data normalized to β-actin show that muscle DJ-1 levels were significantly higher in the exercise group than controls (t-test, **p<0.01). (**B**) DJ-1 levels in plasma were significantly increased in exercise mice compared to control mice (t-test, **p<0.01). (**C**) Brain DJ-1 levels were higher in exercise mice than control mice, but not significantly (t-test, *p* = 0.06). (**D**) Correlations between muscle and plasma DJ-1 (dashed line) and brain DJ-1 (solid line) in exercising mice. Both plasma and brain DJ-1 levels were significantly correlated with the exercise-induced change in muscle DJ-1 (plasma vs. muscle, *p* = 0.04 and brain vs. muscle, *p* = 0.03). All DJ-1 values are relative to non-exercise controls.

6-month old FVB/N wild-type mice were individually housed with running wheels mounted in each cage. Controls had locked running wheels. The daily running distances were recorded using an odometer. In less than 24 hours, mice started spontaneous exercise in the running wheels. Running distances reached a steady-state rate within 48 hours of access to the wheels. Average daily distance was 2.88 ± 0.59 miles (n = 5). After one week of exercise, mice were sacrificed, and brain, muscle, and blood were collected for analysis. Since we have previously reported that higher DJ-1 levels in brain are associated with reduced α-synuclein aggregation, we examined DJ-1 levels in mouse tissues and plasma after exercise. Using Western blots and ELISA assays, we found that running mice had significantly higher DJ-1 levels in muscle (Fig 1**A**, **p<0.01) and in plasma (Fig 1**B**, **p<0.01) compared to non-exercising control mice. Brain DJ-1 levels reached near-significance in exercising mice compared to non-exercising controls (Fig 1**C**, *p* = 0.06). When the levels of DJ-1 were correlated between muscle, plasma, and brain, we found positive correlations in DJ-1 levels between muscle and plasma (*p* = 0.04, Fig 1**D**, dashed line), and also between muscle and brain (*p* = 0.03, Fig 1**D**, solid line). The positive correlations demonstrate that mice which had higher DJ-1 levels in muscle also had higher DJ-1 levels in plasma and brain.

### Running wheel and Rotarod performance in DJ-1 knockout mice

**Fig 2:**
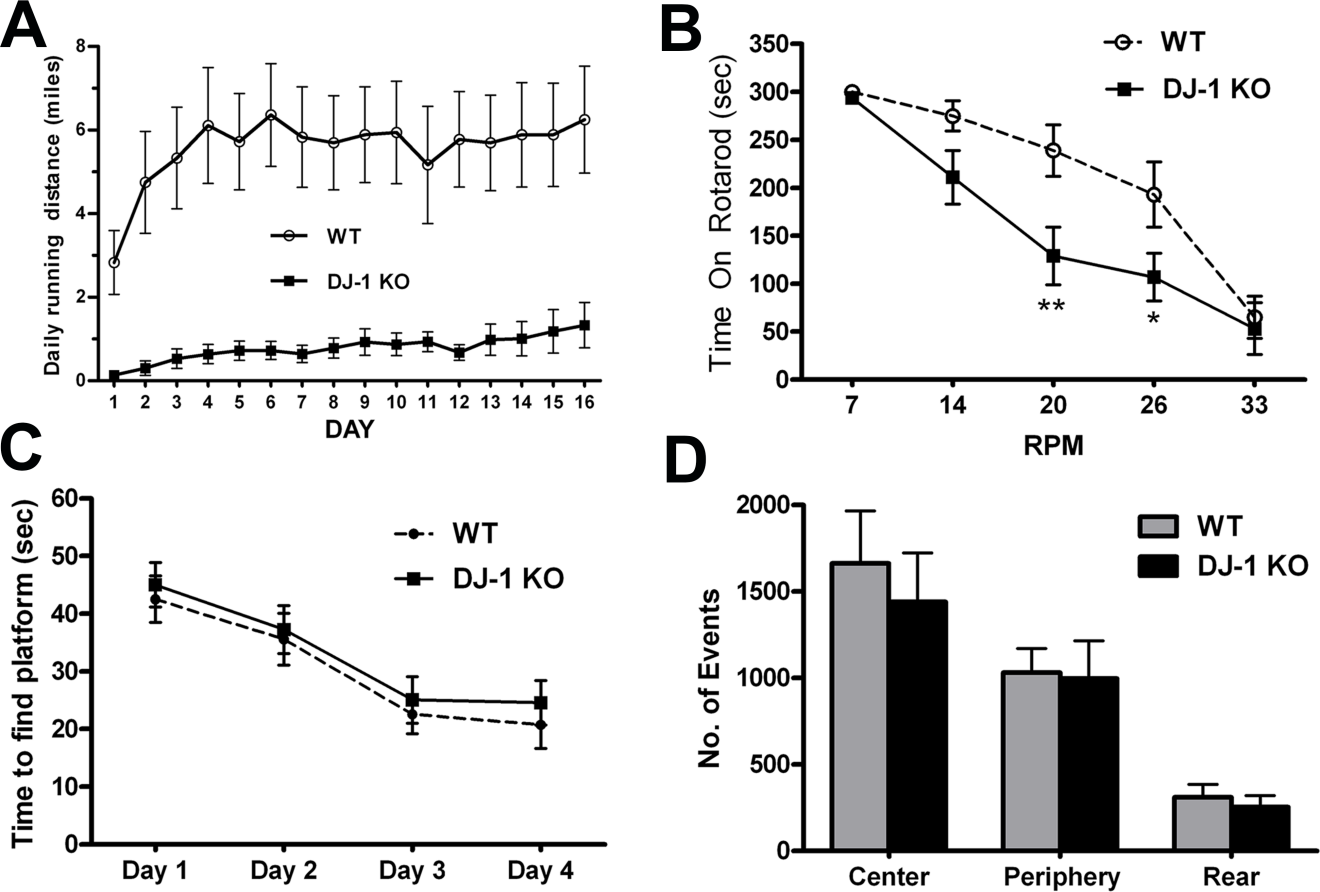
DJ-1 knockout mice had impaired performance on running wheels and on the Rotarod but normal cognitive and exploratory activity. Ten months old homozygous DJ-1 knockout (DJ-1 KO) mice and C57BL/6 wild-type (WT) littermates were trained to perform running wheel exercise in their individual cages. Daily running distances were recorded for two weeks. (**A**) Wild-type mice ran 5.57 ± 0.21 miles per day, while DJ-1 knockout mice were significantly slower, running 0.89 ± 0.06 miles per day (n = 6, multi-variance ANOVA test, **p<0.01). Ten months old DJ-1 knockout mice and C57BL/6 wild-type littermates were also tested on the Rotarod (**B**), Morris water maze (**C**), and open field (**D**). (**B**) DJ-1 knockout mice fell from the Rotarod after a shorter period of time than WT mice when tested at 20 and 26 rpm (n = 6, multi-variance ANOVA, F_(4, 60)_ = 16.93, *p* = 0.0089, *p<0.05, **p<0.01). (**C**) DJ-1 knockout mice and WT littermates had similar learning curves in the Morris water maze (n = 6, multi-variance ANOVA, *p*>0.1). (**D**) DJ-1 knockout mice and WT mice had similar exploratory activity in open field testing (n = 6, multi-variance ANOVA, *p*>0.1).

To study the importance of the DJ-1 gene in sustained, voluntary performance on a running wheel, we tested mice which had the DJ-1 gene knocked out. We compared 10-month old homozygous DJ-1 knockout mice with same age wild-type C57BL/6 littermates for their running ability over a 16-day period (n = 6, each group). Both groups learned to use the running wheels in the first three days, and their running distances reached a stable level after 6 days. We found that wild-type C57BL/6 mice ran 5.57 ± 0.21 miles per day. By contrast, the DJ-1 knockout mice had significantly lower running wheel performance, only 0.89 ± 0.06 miles per day (p<0.01), as shown in Fig 2**A**.

To evaluate the effects of DJ-1 gene deletion on high-speed motor activity and on cognitive function, we performed Rotarod, Morris water maze, and open field tests in 10-month old DJ-1 knockout mice and age-matched wild-type littermates. We found that DJ-1 knockout mice fell off the Rotarod sooner than wild-type littermates at settings of 20 rpm and 26 rpm (Fig 2**B**, ANOVA F_(4, 60)_ = 16.93, *p* = 0.0089; **p<0.01 at 20 rpm, *p<0.05 at 26 rpm), indicating motor deficits in DJ-1 knockout mice. By contrast, DJ-1 knockout mice had normal cognitive function as assessed in the Morris water maze since both knockout and wild-type animals had similar learning curves to find the hidden platform during four days of training (Fig 2**C**). DJ-1 knockout mice had exploratory activity in open field testing that was the same as their wild-type littermates (Fig 2**D**).

### Effects of long term running wheel exercise on motor and cognitive function in transgenic mice with Y39C mutant human α-synuclein

**Fig 3:**
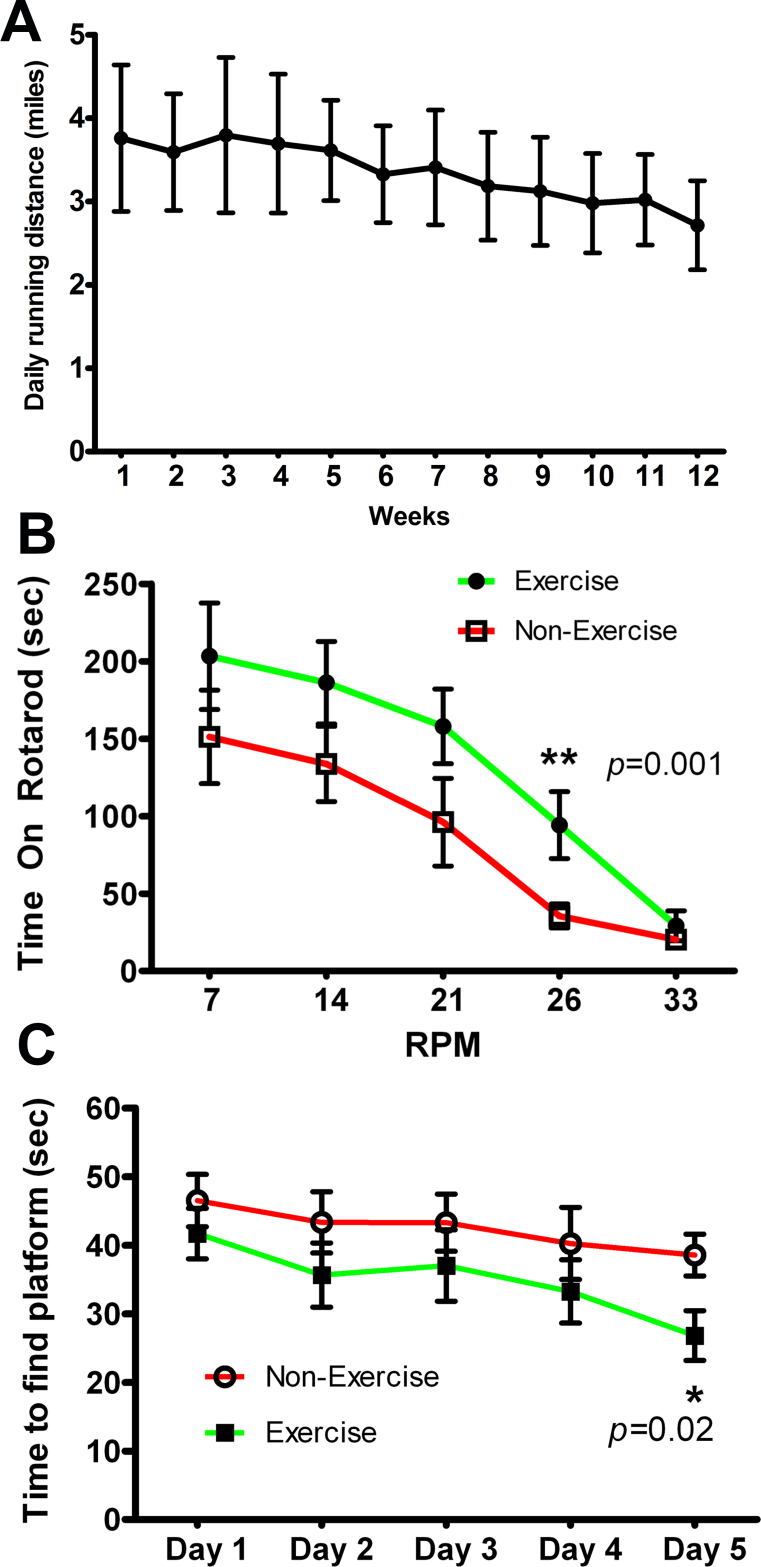
Y39C human mutant α-synuclein transgenic mice performed daily running wheel exercise for three months which led to improved motor and cognitive function. 12-month-old Y39C transgenic mice were divided into Exercise and Non-Exercise groups (n = 7 for each group) following pre-testing of all 14 animals in individual cages with running wheels. Animals were assigned to Exercise and Non-Exercise groups by alternating rank order following their week-long pre-test. Exercise mice had free access to individual cage-mounted running wheels and Non-Exercise mice had a locked, non-functioning running wheel in individual cages. Daily running distances of the Exercise animals were recorded and averaged for each week. (**A**) Data show that all animals continued running for 12 weeks with some reduction in running speed. Average distance in the first week was 3.76 ± 0.87 miles per day. Average distance in the 12^th^ week was 2.71 ± 0.53 miles per day (no statistical difference between 1^st^ and 12^th^ week, n = 7, multi-variance ANOVA, *p* = 0.33). After 12-weeks of running wheel activity, all mice were tested for high intensity motor activity on the Rotarod (**B**) and cognitive function using a Morris water maze (**C**). (**B**) In the Rotarod test, the Exercise group could remain on the rod significantly longer at 26 rpm than the Non-Exercise group (n = 7, multi-variance ANOVA, **p = 0.001). (**C**) In the Morris water maze, the Exercise mice took significantly less time to find the hidden platform at Day 5 than Non-Exercise transgenic mice (n = 7, multi-variance ANOVA, *p = 0.02).

After demonstrating the importance of the DJ-1 gene and protein during voluntary and forced motor activity, we then explored long term running wheel exercise on the age-related motor and cognitive decline and neuropathology seen in transgenic mice expressing mutant human α-synuclein. We have previously described transgenic mice expressing Y39C human α-synuclein in all neurons under control of the Thy1 promoter. These animals develop progressive, age-related α-synuclein protein aggregation and behavioral dysfunction starting at 12 months of age [56]. To study the effects of exercise on development of behavioral and brain tissue pathology, 12-month old Y39C animals were tested on running wheels for one week to evaluate their baseline running activity (n = 14). Running distances were recorded over a 7-day period, and animals were ranked from fastest to slowest. Mice were alternately assigned in rank order to either Exercise or Non-Exercise groups (n = 7, each group). Animals were individually housed with the Exercise group having a functional running wheel in each cage. The Non-Exercise mice had locked running wheels. For the exercise mice, running distances were recorded for each week and converted to daily distances for each mouse (Fig 3**A**). Results after 12 weeks showed that all transgenic mice with functioning running wheels continued to run long distances without significant decline for the group as a whole. Average distance in Week 1 was 3.76 ± 0.87 miles per day, and was 2.71 ± 0.53 miles per day by Week 12. This apparent reduction was not significantly different from the initial running rate (*p* = 0.33).

To determine whether running wheel activity could improve high speed motor performance, we used a Rotarod test. After 12 weeks, we found that the Exercise group had significantly better performance on the Rotarod than the Non-Exercise group (Fig 3**B**, **p = 0.001, n = 7 per group). Cognitive function was tested in the Morris water maze. After 12 weeks of exercise, we found that the exercise group located the hidden platform significantly faster than the non-exercise group on Day 5 of testing (Fig 3**C**, *p = 0.02, n = 7 each group).

### Exercise-induced changes in DJ-1, Hsp70 and BDNF levels in brain, muscle, and plasma of mutant α-synuclein transgenic mice

**Fig 4:**
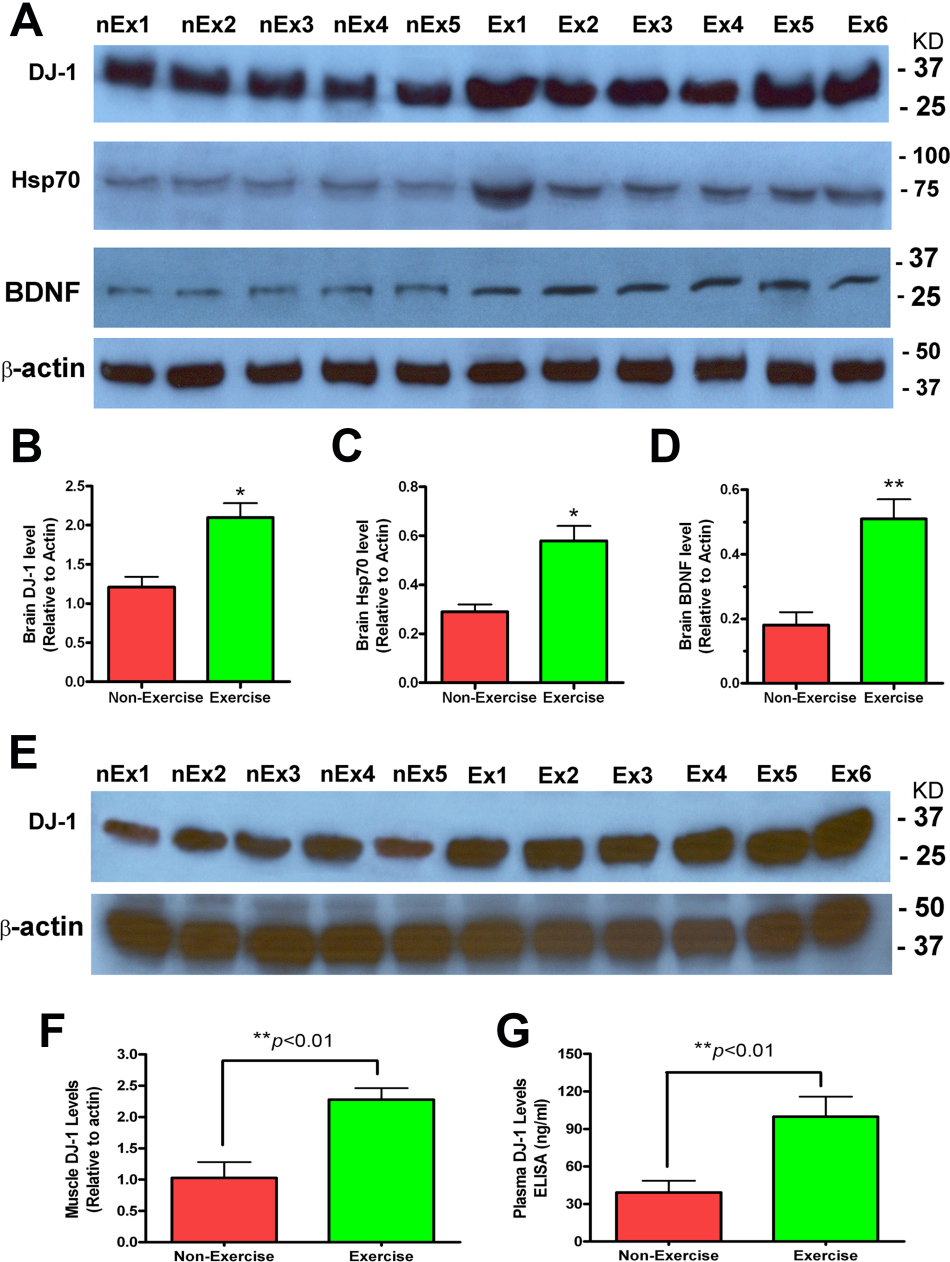
Exercise increased DJ-1, Hsp70 and BDNF levels in 15-month old Y39C transgenic mouse brain and also increased DJ-1 levels in muscle and plasma. (**A**) Brain tissues (cortex) were processed for Western blot analysis using antibodies to DJ-1, Hsp70, BDNF and β-actin after 3 months of running wheel Exercise (**Ex**) or no Exercise (**nEx**) in Y39C transgenic animals. Representative images are shown for all Western blots. (**B-D**) Quantitative protein levels in brain are shown for each group after being normalized to β-actin. There were significant increases in DJ-1, Hsp70 and BDNF proteins in Exercise mouse brain compared to Non-Exercise mice (n = 7, t-test, *p<0.05, **p<0.01). (**E)** Muscle samples were processed for Western blot analysis using DJ-1 and β-actin antibodies after 3 months of running wheel Exercise (**Ex**) or no Exercise (**nEx**) in control animals. (**F**) Muscle DJ-1 Westerns were quantified and are shown for each group after being normalized to β-actin. Muscle DJ-1 was significantly increased in Exercise mice compared to Non-Exercise mice (n = 7, t-test, **p<0.01). (**G**) Plasma DJ-1 levels were measured by ELISA. Results show that the Exercise group had significantly higher plasma DJ-1 concentrations than the Non-Exercise mice (n = 7, t-test, **p<0.01).

Using Western blot analysis, we looked for biochemical changes in the tissue samples of the 15-month old Y39C-transgenic mice after 3 months of running wheel exercise. Brain DJ-1 levels were significantly increased in the exercise group compared to the non-exercise group (Fig 4**A** and 4**B**, *p<0.05, n = 7, both groups). Brain Hsp70 levels were significantly higher in the brains of mice which exercised compared to the non-exercising mice (Fig 4**A** and 4**C**, *p<0.05, n = 7). The neurotrophic factor BDNF was also increased in exercising mice (Fig 4**A** and 4**D**, *p<0.05, n = 7).

DJ-1 protein levels were measured in muscle and plasma of the 15-month old transgenic mice. Results from muscle using Western blots showed that exercising mice had significantly higher DJ-1 levels than non-exercising mice (Fig 4**E** and 4**F**, *p<0.01, n = 7). For analysis of plasma DJ-1, we used ELISA assays. We found that plasma DJ-1 levels were more than doubled in the Exercise group compared to the Non-Exercise group (Fig 4**G**, **p<0.01, n = 7).

### Effects of running wheel exercise on α-synuclein aggregation in brain of transgenic mice

**Fig 5:**
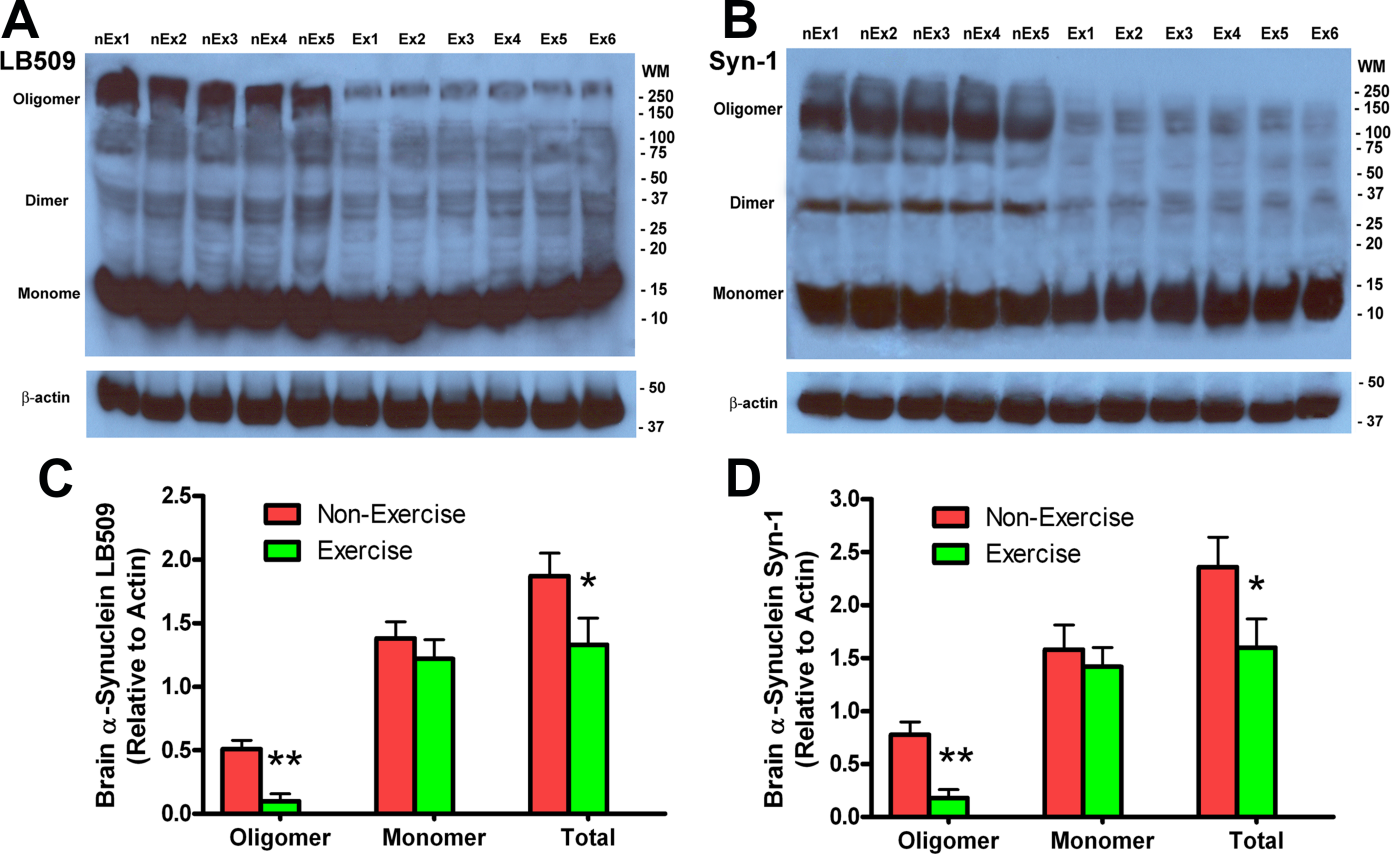
Exercise reduced α-synuclein oligomer formation in 15 month-old Y39C transgenic mouse brain. Brain tissues (cortex) from Exercise and Non-Exercise transgenic mice were analyzed for α-synuclein aggregation using LB509 (human only) and Syn-1 (mouse-plus-human) antibodies. (**A**) Western blots with LB509 show that exercise dramatically reduced α-synuclein oligomer formation in brain compared to mice of the same age not performing exercise. α-Synuclein monomer levels were not changed. The sample blots show Exercise (**Ex**) and Non-Exercise (**nEx**) animals. (**B**) Western blots with Syn-1 antibody show that Exercise mice had reduced α-synuclein oligomer and dimer fractions, as compared to Non-Exercise mice. (**C, D**) Western blot images were quantified. The relative levels of α-synuclein oligomer, monomer, and total (oligomer plus monomer) to β-actin are shown for Exercise and Non-Exercise transgenic mice. There were significant reductions in brain oligomer (n = 7, t-test, **p<0.01) and total (n = 7, t-test, *p<0.05) in the Exercise group compared to the Non-Exercise group.

The key neuropathological feature of Y39C transgenic mice is the progressive accumulation of α-synuclein protein in neurons [56]. To see if running wheel exercise can reduce α-synuclein deposition, we performed Western blot analysis on the 15-month old transgenic mouse brain tissue. We used two different antibodies to α-synuclein: LB509 which recognizes human α-synuclein only and Syn-1 which detects both human and endogenous mouse α-synuclein. In Western blots with LB509 antibody, we found that mice which exercised had significantly reduced human α-synuclein oligomer formation in brain compared to age-matched transgenic mice which did not exercise (Fig 5**A** and 5**C**, **p<0.01, n = 7). We noted that human α-synuclein monomer was not reduced. *Total* human α-synuclein was significantly reduced in exercising mice because of the lower oligomer concentration (Fig 5**A** and 5**C**, *p<0.05, n = 7). Similarly, using Syn-1 antibody in Western blots, we found that mice which exercised had significantly less human-plus-mouse α-synuclein oligomer formation in brain than seen in age-matched, non-exercising transgenic mice (Fig 5**B** and 5**D**, **p<0.01, n = 7). *Total* human-plus-mouse α-synuclein was also significantly reduced in exercising transgenic mice (Fig 5**B** and 5**D**, *p<0.05). Human-plus-mouse monomer concentrations were not changed. These results indicate that running wheel exercise can prevent α-synuclein aggregation in brain by reducing oligomer formation.

### Effects of running wheel exercise on α-synuclein in plasma of transgenic mice

**Fig 6:**
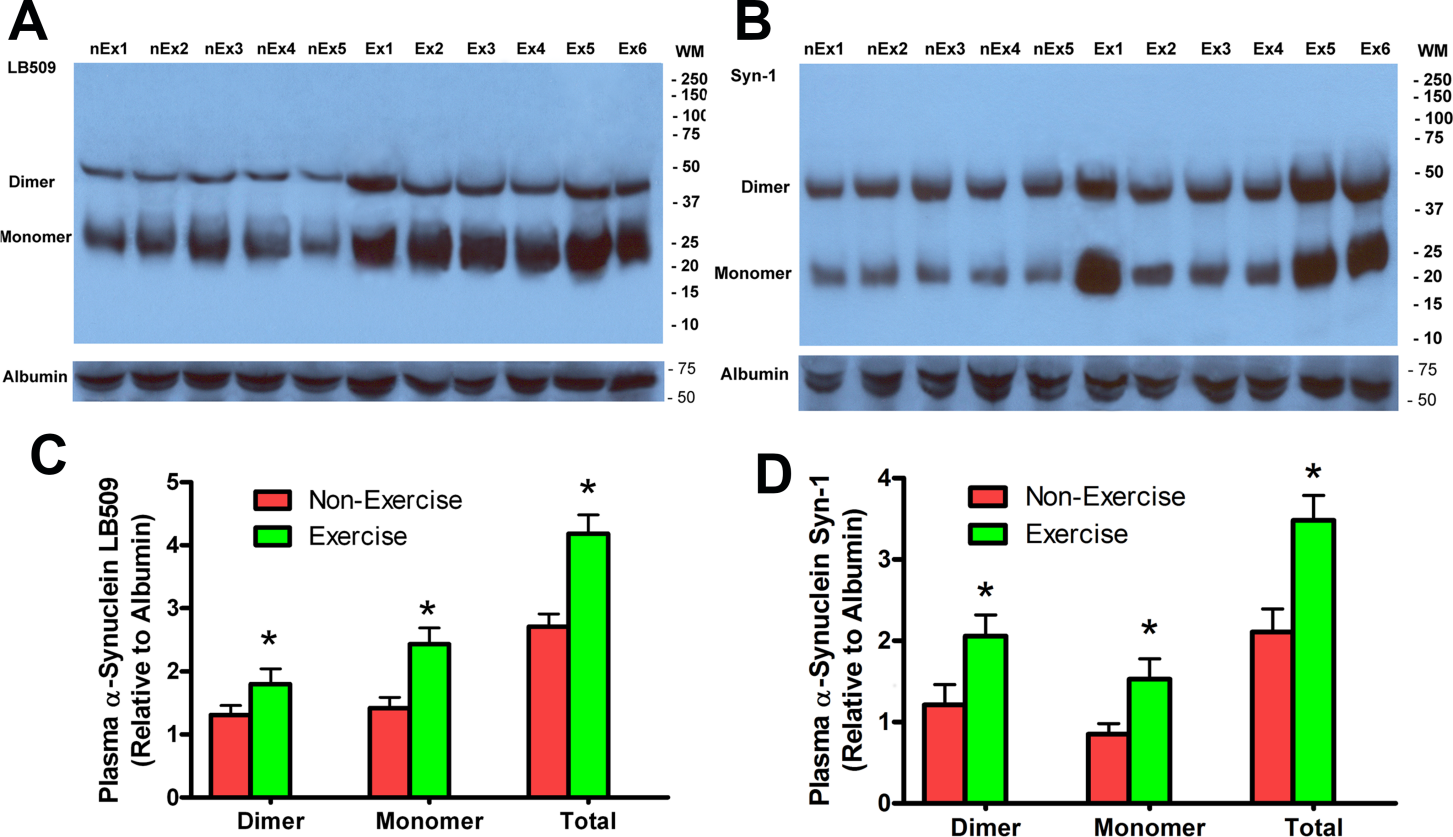
Exercise increased α-synuclein concentration in blood plasma in 15-month old Y39C transgenic mouse. Mouse blood plasma samples were processed by Western blot analysis using antibodies to LB509 (**A**) and Syn-1 (**B**). Both monomer and dimer α-synucleins were found in mouse plasma. Sample images are shown for each Western blot. (**C, D**) Quantitative α-synuclein plasma levels are shown for each group after being normalized to mouse serum albumin. With both LB509 (**A**) and Syn-1 (**B**) antibodies, there were significant increases in α-synuclein monomer, dimer and total (monomer plus dimer) fractions in Exercise versus Non-Exercise mouse plasma (n = 7, t-test, *p<0.05).

While α-synuclein is a neuronal protein concentrated in the synapse and is not present in muscle, α-synuclein is found in plasma. Red blood cells contain α-synuclein, although its biological function in those cells is unknown [57]. We have found that transgenic mouse red blood cells contain only mouse α-synuclein while plasma α-synuclein is a combination of human and mouse protein in a proportion similar to that seen in transgenic mouse brain [56]. This result indicates that plasma α-synuclein has originated from neurons expressing both wild-type α-synuclein and mutant human α-synuclein. We examined whether exercise can modify α-synuclein plasma levels. Using Western blots with the LB509 antibody, we found that exercising mice had significantly higher plasma levels of human α-synuclein in both monomer and dimer fractions (Fig 6**A** and 6**B**, *p<0.05, n = 7). Similarly, using the Syn-1 antibody we found that the exercise group had significantly increased α-synuclein monomers and dimers in blood plasma compared to non-exercising transgenic mice (Fig 6**C** and 6**D**, *p<0.05, n = 7). Because exercise reduces brain α-synuclein while increasing plasma α-synuclein, this result indicates that exercise enhances the clearance of α-synuclein from brain into the bloodstream.

## Discussion

We have discovered that a functional DJ-1 gene is required for normal, voluntary running wheel performance in mice. In young wild-type mice as well as in aging transgenic mice expressing mutant human α-synuclein in all neurons, running wheel exercise can increase DJ-1 protein levels in muscle, plasma, and brain. We have found that long term running wheel exercise has a neuroprotective effect in our transgenic mice. Exercise significantly improves motor and cognitive function while dramatically reducing α-synuclein oligomer accumulation in brain while increasing plasma concentrations of α-synuclein. The mechanism by which exercise leads to these beneficial effects appears to be related to upregulation of DJ-1 and other neuroprotective factors such as Hsp70 and BDNF in the brain.

We and others have reported that increased expression of DJ-1 can render neurons more resistant to oxidative stress and to misfolded protein accumulation [34, 58, 59]. Using *in vitro* experiments in N27 dopamine neurons, we have demonstrated that increased DJ-1 protein levels can protect neurons from oxidative stress by increasing glutathione production through upregulation of the rate limiting step in glutathione synthesis. If, instead, the cell stress is overexpression of mutant human α-synuclein, increased levels of DJ-1 do not change glutathione synthesis but do increase expression of Hsp70 [34]. In the current study, the exercise-induced increase in DJ-1 and Hsp70 in brain are likely preventing the formation of α-synuclein oligomers. Other researchers have shown that exercise can increase neurogenesis in hippocampus, increase BDNF expression, and improve memory function in various mouse models [60–65]. Treadmill running can activate the anti-oxidant master gene Nrf2 and protect mice from the neurotoxic effects of MPTP [40, 44, 66]. In the Alzheimer transgenic mouse model, exercise can decrease β-amyloid concentrations by the activation of SIRT-1 signaling pathway [50]. There has been no previous investigation of long-term exercise in transgenic models of Parkinson’s disease. Our results indicate that long-term exercise can prevent the development of age-related neurodegeneration in a transgenic mouse model of diffuse Lewy body disease, a Parkinson-Plus disorder. In humans, diffuse Lewy body disease is a currently untreatable form of Parkinson’s.

Because exercise produces sweeping changes in all aspects of physiology from sensorimotor activity to lipid metabolism in muscle, it is difficult to define a hierarchy of beneficial effects on brain function. Since mice which lack the DJ-1 gene cannot perform on running wheels or on the Rotarod with the same intensity as wild-type animals, DJ-1 appears to be essential for dealing with the physiological stress created in muscle by sustained motor activity. Because DJ-1 knockout animals have the same cognitive performance as wild-type mice in the Morris Water Maze and on open field exploration, the DJ-1 deficit does not appear to influence cognition nor low intensity motor activity. To precisely define the role of muscle verse brain derived DJ-1, organ-specific DJ-1 knockouts would have to be developed.

Our study gives insight into the mechanism by which exercise prevents α-synuclein oligomer accumulation in brain. While oligomer formation was reduced in brains of mice with access to running wheels, the same animals showed increased plasma concentrations of α-synuclein monomers and dimers. α-Synuclein is known to be present in plasma of humans and other mammals, but the exact source of plasma α-synuclein remains uncertain. While it is possible that red blood cells may release α-synuclein into plasma, the protein may come from central and peripheral neurons [67, 68]. Our findings in Y39C transgenic mice show that plasma α-synuclein comes from neurons rather than red blood cells because plasma α-synuclein is approximately 50:50 human/mouse mixture as is brain. By contrast, red blood cell α-synuclein is 100% mouse in our Y39C animals.

α-Synuclein has been found in plasma exosomes as a soluble protein [69, 70]. Studies have shown that α-synuclein can be secreted from cultured neurons via exosomes [71, 72]. We have recently shown that lysosomal and exosomal genes are activated *in vitro* by overexpression of mutant human α-synuclein as well as by DJ-1. Activated lysosomes and exosomes are the likely route of enhanced α-synuclein secretion from the cytosol to the plasma (Cummiskey, Zhou, Freed et al., unpublished data). In the same transgenic mouse strain used in the current study, we have shown that the drug phenylbutyrate can increase DJ-1 levels, prevent α-synuclein oligomer formation in brain, and stop age-related decline in motor and cognitive function [35]. Exercise appears to have the same neuroprotective effects as phenylbutyrate. Our earlier *in vitro* experiments showed that the beneficial effects of phenylbutyrate were dependent on the expression of the DJ-1 gene. Blocking DJ-1 expression with anti-sense RNA blocked the drug’s ability to protect neurons from oxidative stress and from misfolded protein. This result indicates that DJ-1 is required to exert the neuroprotective effect of phenylbutyrate [35]. Because we have found that DJ-1 knockout mice have much reduced spontaneous activity on running wheels and have impaired performance on the Rotarod, the DJ-1 protein appears to be essential for normal motor function. Since DJ-1 knockout animals have normal swimming and cognitive abilities in the Morris Water maze as well as normal exploratory behavior in open field testing, it is likely that their reduced activity on the running wheel or the Rotarod represents a limitation in maximal muscle performance rather than in cognitive function.

In summary, we have found that voluntary exercise on a running wheel can upregulate DJ-1 in muscle and brain of a transgenic mouse model of Parkinson’s disease and can prevent the age-related decline of motor and cognitive abilities normally seen in this transgenic strain. Since we have described similar beneficial effects with the drug phenylbutyrate in these transgenic mice, we hypothesize that patients with Parkinson’s disease might be able to slow or stop disease progression from either an intensive exercise program or treatment with the drug phenylbutyrate.

## Abbreviations

BDNF: brain derived neurotrophic factor
Ex: exercise
Hsp70: heat shock protein 70
LB509: antibody specific for human α-synuclein
nEX: non-exercise
RPM: revolutions per minute
Syn-1: antibody recognizing both mouse and human α-synuclein
Tg: transgenic
Y39C: tyrosine replaced by cysteine at residue 39 of human α-synuclein
